# Syn3, a newly developed cyclic peptide and BDNF signaling enhancer, ameliorates retinal ganglion cell degeneration in diabetic retinopathy

**DOI:** 10.1093/procel/pwae028

**Published:** 2024-05-14

**Authors:** Ke-ran Li, Meng-Jia Huan, Jin Yao, Jia-jun Li, Yuan Cao, Suyu Wang, Mandar T Naik, Yuan Fang, John Marshall, Chang-gong Lan, Cong Cao

**Affiliations:** The Affiliated Eye Hospital, Nanjing Medical University, Nanjing 210029, China; Department of Ophthalmology, The Second Affiliated Hospital of Nanjing Medical University, Nanjing 210029, China; The Affiliated Eye Hospital, Nanjing Medical University, Nanjing 210029, China; The Affiliated Eye Hospital, Nanjing Medical University, Nanjing 210029, China; The Affiliated Eye Hospital, Nanjing Medical University, Nanjing 210029, China; The Affiliated Eye Hospital, Nanjing Medical University, Nanjing 210029, China; Department of Molecular Pharmacology, Physiology and Biotechnology, Brown University, Providence, RI 02912,United States; Department of Ophthalmology & Vision Science, Eye and ENT Hospital, Shanghai Medical College, Fudan University, Shanghai 200433, China; Department of Molecular Pharmacology, Physiology and Biotechnology, Brown University, Providence, RI 02912,United States; Department of Joint Surgery and Geriatric Orthopedics, Affiliated Hospital of YouJiang Medical University for Nationalities, Guangxi Key Laboratory of Basic and Translational Research of Bone and Joint Degenerative Diseases, Guangxi Biomedical Materials Engineering Research Center for Bone and Joint Degenerative Diseases, Baise 533000, China; Clinical Research Center of Neurological Disease, The Second Affiliated Hospital of Soochow University, Institution of Neuroscience, Soochow University, Suzhou 215031, China

**Dear Editor,**

Diabetic retinopathy (DR) remains a leading cause of irreversible blindness in adults worldwide (Sabanayagam et al., 2019). Increasing evidence suggests that visual deficits are closely associated with neurodegeneration, especially the synaptic loss and degeneration of retinal ganglion cells (RGCs) (Kern and Barber, 2008). Neurotrophic factors play a key role in supporting the survival of RGCs (Kimura et al., 2016) and brain-derived neurotrophic factor (BDNF) stands out as a crucial neurotrophin vital for RGCs (Kimura et al., 2016). *In vivo* studies have demonstrated that intravitreal administration of BDNF enhances the survival of axotomized RGCs in rats, and astrocytes designed to secrete BDNF promote RGC survival *in vitro* (Castillo et al., 1994). The levels of BDNF in both serum and aqueous humor are significantly reduced in patients with diabetes mellitus prior to the onset of clinical signs of retinopathy (Taslipinar Uzel et al., 2020). In streptozotocin (STZ)-injected DR rats, *BDNF* mRNA, and protein expression is reduced in retinal tissues (Seki et al., 2004) and retinal neuropathy was ameliorated by intraocular administration of BDNF (Seki et al., 2004). These findings demonstrate the physiological significance of reduced BDNF signaling in the development of DR and the degeneration of RGCs, suggesting that bolstering BDNF signaling may offer neuroprotection to RGCs in diabetes (Seki et al., 2004).

Upon BDNF stimulation, the tropomyosin receptor kinase B (TrkB) receptor associates with the adaptor proteins Gab1, SHP2, and Grb2, which facilitate the initiation of the phosphoinositide 3-kinase-Akt-mammalian target of rapamycin (mTOR), phospholipase C, and the Ras/MAPK intracellular signaling pathways (Kimura et al., 2016). Postsynaptic density protein-95 (PSD95) is a synaptic scaffolding protein involved in the trafficking and stabilization of N-methyl-d-aspartate receptors and α-amino-3-hydroxy-5-methyl-4-isoxazolepropionic acid receptors at the postsynaptic membrane to regulate glutamatergic transmission and synaptic plasticity (Cao et al., 2013; Marshall et al., 2018). Our group (Cao et al., 2013; Lau et al., 2023) and others (Ji et al., 2005) identified PSD95 as a TrkB-associated scaffolding protein required for intact downstream PLC and Akt-mTOR signaling (Cao et al., 2013).

Intravitreal delivery of BDNF has been shown to mitigate RGC degeneration following optic nerve injury, although its efficacy is limited due to TrkB downregulation (Fudalej et al., 2021). Treatment with BDNF is also problematic due to potential toxicities arising from its activation of the p75NTR pathway and the possible truncated TrkB.T1 isoform implicated in retinal degeneration (Yanpallewar et al., 2012). In an attempt to circumvent these limitations, we developed a series of peptidomimetic compounds that bind PSD95 to facilitate its association with TrkB and enhance BDNF signaling (Cao et al., 2013; Lau et al., 2023). One of these compounds CN2097 (R_7_-CC-YK[KTE(β-Ala)]V) consists of a cyclic moiety based on CRIPT, incorporating a β-alanine lactam side chain linker between the valine and threonine residues that binds both the PDZ1 and PDZ3 domains of PSD95 and a poly-arginine (R_7_) blood–brain barrier active-transport moiety (Cao et al., 2013; Lau et al., 2023). In Angelman syndrome, where BDNF signaling is impaired (Cao et al., 2013), CN2097 enhanced BDNF signaling to restore long-term potentiation and mitigate neurological deficits (Cao et al., 2013; Lau et al., 2023). In this study, we first investigated the efficacy of CN2097 in the treatment of DR. Finding that CN2097 administration potently alleviated RGC degeneration, we next tested the efficacy of a newly developed cyclic peptide, named Syn3, which specifically binds the PDZ3 domain of PSD95 with nanomolar affinity (Naik et al., 2024). We found that Syn3 boosts the formation of the TrkB-PSD95-Gαi1/3 complex, and is more potent than CN2097 in providing robust protection for RGCs in DR model mice.

We first assessed the ability of CN2097 to promote BDNF signaling in primary murine RGCs. Pretreatment with CN2097 (2 μmol/L) for twenty minutes significantly enhanced BDNF-induced Akt and S6K phosphorylation (*P* < 0.001 vs. BDNF-only treatment, Fig. S1A), without affecting TrkB phosphorylation (*P* > 0.05 vs. BDNF-only treatment, Fig. S1A) or the expression of TrkB, Akt1, and S6K (Fig. S1A). To determine whether CN2097 protects RGCs *in vivo*, the streptozotocin (STZ)-induced DR mouse model was established. CN2097 was intravitreously administered at day 42 and day 56 after the final STZ administration (Fig. S1B). In DR mice (10 weeks after the last STZ administration), the number of nuclei in the retinal ganglion cell layer (GCL) was decreased by 45.08% ± 9.54% compared the vehicle-administrated control mice (“Ctrl,” *P* < 0.001, nice mice per group, Fig. S1C). Significantly, CN2097 administration ameliorated RGC degeneration in DR mice (12.22 ± 2.59 vs. 22.01 ± 2.80 per view, *P* < 0.001, Fig. S1C), quantifying nuclei from Hematoxylin and Eosin (HE)-stained retinal sections. Fluorescence staining of retinal sections confirmed that the number of NeuN-positive RGCs in the GCL was dramatically decreased in the DR mice (47. 8% ± 9.92% compared to Ctrl mice, *P* < 0.001, Fig. S1D). CN2097 administration reduced RGC degeneration in DR mice (10.89 ± 2.526 vs. 15.56 ± 3.88 per view, *P* < 0.01, Fig. S1D). Furthermore, the expression levels of the RGC marker proteins Thy-1 (a surface glycoprotein uniquely expressed on RGCs) and β3-tubulin showed a significant decrease in the retinal tissues of STZ-administrated DR mice (quantified from retinal tissues of six mice per group, *P* < 0.001 vs. control mice, Fig. S1E). Notably, administration of CN2097 potently mitigated the downregulation of Thy-1 and β3-tubulin proteins (*P* < 0.001 vs. DR mice, Fig. S1E).

CN2097 binds the PSD95 with relatively low affinity (Naik et al., 2024). Additionally, although the cyclic peptide was based on the C-terminal sequence of CRIPT which specifically binds the PDZ3 domain, we surprisingly found that CN2097 also interacts with the PDZ1 domain, which could reduce efficacy (Naik et al., 2024). A model of CN2097 bound to PDZ3 domain is shown in Fig. 1A.

**Figure 1.**
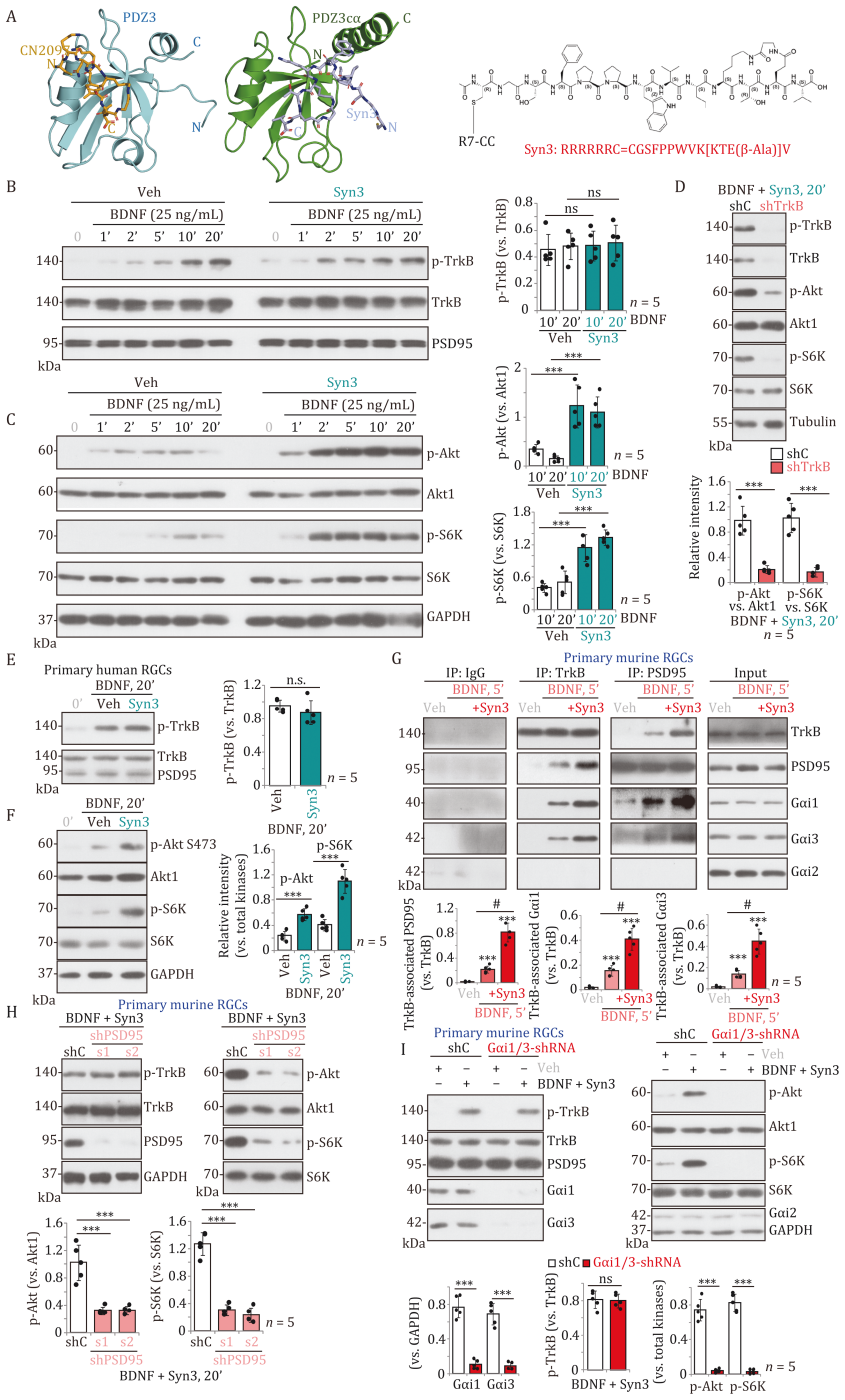
**Syn3 enhances BDNF-TrkB signaling in primary RGCs.** Experiment guided models of CN2097 interaction with PSD95 PDZ3 domain and Syn3 interaction with the longer PDZ3cα domain. These models were built using sparse NMR chemical shift perturbation data using software Haddock version 2.4. (A). The amino acid structure/sequence of Syn3 is also presented (A). The primary murine or human RGCs were pretreated with Syn3 (0.2 μmol/L) or vehicle control (saline, “Veh”) for 20 min, followed by BDNF stimulation (25 ng/mL) for listed time, expression of listed proteins in total cell lysates was tested (B, C, E, and F). The primary murine RGCs with the lentiviral TrkB shRNA (“shTrkB”) or the lentiviral scramble control non-sense shRNA (“shC”) were treated with BDNF (25 ng/mL) plus Syn3 (0.2 μmol/L, 20 min pretreatment) (“BDNF + Syn3”) for 20 min and were tested for the listed proteins in total lysates (D). The primary murine RGCs were pretreated with Syn3 (0.2 μmol/L) or vehicle control (saline, “Veh”) for 20 min, followed by BDNF stimulation (25 ng/mL) for 5 min, TrkB-PSD95-Gαi1/3 association was tested by co-immunoprecipitation (Co-IP) assays (G), and their expression examined (in “Input,” G). The primary murine RGCs with the listed lentiviral PSD95 shRNA (“shPSD95-s1” or “shPSD95-s2”) or the lentiviral scramble control shRNA (“shC”) were treated with BDNF + Syn3 and were analyzed for the listed proteins in total cellular lysates (H). The primary murine RGCs with the lentiviral Gαi1 shRNA plus lentiviral Gαi3 shRNA (Gαi1/3-shRNA) or the lentiviral scramble control shRNA (shC) were treated with BDNF + Syn3 or vehicle control (saline, “Veh”) and were analyzed for the listed proteins in total cellular lysates (I). Data were presented as mean ± standard deviation (SD). *n* = 5 stands for five biological repeats. ***P < 0.001. ***P < 0.001 vs. “Veh” (G). ^#^*P* < 0.05 (G). “ns” stands for non-statistical difference (*P* > 0.05).

To develop a compound that binds the PDZ3 domain with higher affinity we took advantage of a unique αC helix located after the PDZ3 domain (Naik et al., 2024). A novel peptidomimetic, Syn3, was designed that fused the αC helix-interacting residues derived from SynGAP with the CRIPT-cyclic peptide PBM, which increased PDZ3 affinity by five-fold compared to CN2097 (Naik et al., 2024). Surface plasmon resonance results show that Syn3 has significantly higher on-rates (ka) as well as off-rates (kd) than CN2097 (Naik et al., 2024). A model of Syn3 bound to a longer PDZ3cα domain illustrates the binding of SynGAP-derived residues with the αC helix (Fig. 1A). Despite both compounds containing the CRIPT-cyclic peptide, which interacts similarly within the groove formed between the first alpha helix and the second β-strand of the PDZ3 domain, variations exist in their binding orientations (Fig. 1A).

Testing the ability of Syn3 to enhance BDNF signaling in primary murine RGCs, Syn3 significantly increased phosphorylation of Akt (Ser-473) and p70S6 kinase (S6K, Thr-389) (*P *< 0.001 vs. BDNF-only treatment), without affecting TrkB phosphorylation (Fig. 1B and 1C). Notably, Syn3 was effective at one-tenth the concentration (0.2 μmol/L) required by CN2097 to produce equivalent results. The protein expression of TrkB, Akt1, and S6K in the murine RGCs was unchanged by BDNF or BDNF + Syn3 (Fig. 1B and 1C). Significantly, TrkB silencing using a lentivirus-packed shRNA (“shTrkB”) blocked BDNF (25 ng/mL) plus Syn3-induced Akt-S6K phosphorylation (Fig. 1D), demonstrating that the TrkB receptor is required for Syn3-mediated BDNF signaling. Syn3 treatment alone failed to stimulate TrkB (Tyr-515), Akt (Ser-473), and S6K (Thr-389) phosphorylation (Fig. S2A) and also had no effect on insulin or PDGF receptor signaling (Fig. S2B). In the primary murine RGCs, treatment with insulin (1 μg/mL) or PDGF (-AB, 25 ng/mL) significantly increased Akt-S6K phosphorylation, which was not augmented by Syn3 (0.2 μmol/L) (*P* > 0.05 vs. insulin/PDGF single treatment, Fig. S2B). These results validate that compounds targeting the PDZ3 domain of PSD95 efficiently and specifically enhance BDNF-TrkB signaling in primary murine RGCs. In primary human RGCs pretreated with Syn3 (0.2 μmol/L), BDNF-induced Akt-S6K phosphorylation was significantly enhanced (*P* < 0.001 vs. BDNF only treatment, Fig. 1E and 1F), whereas PSD95 protein expression and TrkB phosphorylation were unchanged (Fig. 1E and 1F).

Oxygen glucose deprivation/re-oxygenation (OGD/R) induces neuronal injury via mechanisms believed to mimic neuronal ischemia and hypoxia injury of DR pathology. We exposed primary murine RGCs to OGD for 4 h followed by 36 h of re-oxygenation (OGD/R), resulting in cell death quantified by a decrease in the number of β3-tubulin-stained RGCs (Fig. S2C). OGDR stimulation reduced cell viability (CCK-8 OD, Fig. S2D) and increased lactate dehydrogenase (LDH) release (a marker of cell death, Fig. S2E). Significantly, the decrease in cell count and viability induced by OGD/R, as well as cell death were mitigated by the combined treatment of BDNF (25 ng/mL) and Syn3 (0.2 μmol/L, pretreatment for 20 min) (“BDNF + Syn3”) (Fig. S2C–E). This combination rescued a significantly greater number of RGCs from OGD/R compared to treatment with BDNF alone (*P *< 0.01 vs. BDNF-only treatment, Fig. S2C–E). In confirmation of the CCK-8 and LDH results, OGD/R stimulation was found to induce significant apoptosis activation as evidenced by increased Caspase-3 activity (Fig. S2F), Caspase-3/Caspase-9/Poly (ADP-ribose) polymerase 1 (PARP1) protein cleavage (Fig. S2G), and TUNEL-positive nuclei (Fig. S2H), which was mitigated by BDNF + Syn3 treatment (Fig. S2F–H). The protective effect of combining BDNF and Syn3 was greater than that achieved with BDNF treatment alone (*P *< 0.01, Fig. S2F–H). Treatment with the TrkB inhibitor K252a or the Akt-specific inhibitor MK-2206 blocked the effects of Syn3 on BDNF-TrkB signaling (Fig. S2I) and protection against OGD/R (Fig. S2J–L). Similarly in primary human RGCs, BDNF-induced RGC survival against OGD/R was potentiated by Syn3 (*P* < 0.01 vs. BDNF-only treatment, Fig. S2M).

The results show that Syn3 enhances TrkB-Akt signaling at a 10-fold lower dose compared to CN2097 to protect RGCs in DR mice. As CN2097 acts to promote TrkB-PSD95 association (Cao et al., 2013; Lau et al., 2023), mechanistically the enhanced binding affinity of Syn3 for the PDZ3 domain of PSD95 is predicted to more potently promote TrkB-PSD95 association. Co-immunoprecipitation (Co-IP) results confirmed that Syn3 pretreatment (20 min, 0.2 μmol/L) augmented TrkB-immunoprecipitated PSD95 in primary murine RGCs (Fig. 1G). TrkB and PSD95 protein expression was unchanged by BDNF (or plus Syn3) (Fig. 1G, “Input”). Our previous studies demonstrated the indispensable role of Gαi1 and Gαi3 (Gαi1/3) in transducing BDNF-TrkB signaling in hippocampal neurons (Marshall et al., 2018). Gαi1/3 associated with BDNF-activated TrkB to promote TrkB endocytosis and downstream signaling (Marshall et al., 2018). In murine RGCs, BDNF treatment similarly induced TrkB immunoprecipitation with both Gαi1 and Gαi3 (Fig. 1G), and augmented with Syn3 pretreatment (20 min, 0.2 μmol/L) (Fig. 1G). Gαi2 protein did not associate with TrkB or PSD95 in murine RGCs (Fig. 1G). Importantly, silencing of PSD95 using two lentivirus-packed PSD95 shRNAs (“shPSD95-s1” or “shPSD95-s2,” with non-overlapping sequences), impaired BDNF (25 ng/mL) plus Syn3 (0.2 μmol/L, 20 min pretreatment) (“BDNF + Syn3”)-induced Akt and S6K phosphorylation (Fig. 1H), and compromised BDNF + Syn3-induced RGC neuroprotection against OGD/R (Fig. S3A–C). Similarly, in primary human RGCs, the application of lentivirus-packed PSD95 shRNA (“shPSD95-s1”) downregulated PSD95 (Fig. S3D) and inhibited BDNF + Syn3-induced Akt-S6K activation (Fig. S3D).

To examine the role Gαi1/3 in BDNF signaling and Syn3 function, the expression of Gαi1 and Gαi3 was knocked-down using Gαi1 and Gαi3 shRNA-expressing lentiviruses (Gαi1/3-shRNA) co-added to primary murine RGCs, that resulted in substantial Gαi1 and Gαi3 protein knockdown after six days (Fig. 1I), with Gαi2 protein expression remaining unchanged (Fig. 1I). Significantly, BDNF (25 ng/mL) plus Syn3 (0.2 μmol/L, 20 min pretreatment) (“BDNF + Syn3”)-induced Akt-S6K phosphorylation was blocked by Gαi1/3 knockdown (Fig. 1I). Gαi1/3 silencing did not significantly affect protein expression of TrkB, PSD95, or BDNF + Syn3-induced TrkB phosphorylation (Fig. 1I). Following exposure of Gαi1/3-shRNA in murine RGCs, BDNF + Syn3-induced neuroprotection against OGD/R was abolished (Fig. S4A–C). In contrast, increasing Gαi1 and Gαi3 protein expression augmented the effects of Syn3 on BDNF signaling (Fig. S4D). The lentivirus-packed Gαi1- and Gαi3-expressing constructs were co-added to primary murine RGCs, leading to Gαi1 and Gαi3 protein overexpression (“oeGαi1/3”) after six days. In two oeGαi1/3 RGC selections (“Slc1 and Slc2”), BDNF + Syn3-induced Akt and S6K phosphorylation was further increased, while TrkB expression and phosphorylation was unchanged (Fig. S4D).

We also utilized dominant negative (DN) strategies to interfere with the association of Gαi1/3 with other signaling proteins. In both the DN-Gαi1 and the DN-Gαi3 constructs the conserved Gly (G) residue was substituted with Thr (T) in G3 box, preventing Gαi1/3 association with adaptor proteins. DN-Gαi1 and DN-Gαi3 lentiviruses were co-added to primary murine RGCs for six days, followed by Western blot to confirm expression in murine RGCs (Fig. S4E). In DN-Gαi1/3 murine RGCs, BDNF (25 ng/mL) plus Syn3 (0.2 μmol/L, 20 min pretreatment) (“BDNF + Syn3”)-induced Akt and S6K phosphorylation was inhibited (Fig. S4E) and TrkB-PSD95-Gαi1/3 association was disrupted (Fig. S4F). The Gαi1 shRNA-expressing lentivirus and the Gαi3 shRNA-expressing lentivirus (Gαi1/3-shRNA) were also added to primary human RGCs and two cell selections established, “Slc1” and “Slc2,” in which Gαi1 and Gαi3 were silenced (Fig. S4G). Gαi2, TrkB, PSD95 protein expression, and BDNF + Syn3-induced TrkB phosphorylation were unchanged (Fig. S4G). Importantly, BDNF + Syn3-induced Akt and S6K phosphorylation was inhibited in Gαi1/3-shRNA-expressing primary human RGCs (Fig. S4G).

Using the DR mouse model, we examined the efficacy of Syn3 to mitigate RGC cell death *in vivo.* Syn3 was intravitreously injected at one-tenth of the concentration of CN2097 using the protocol shown in Fig. 2A. In DR mice, 10 weeks after the last STZ administration, HE staining of retinal sections showed a significant decrease in the number of nuclei in the GCL by 57.72% ± 6.28% compared to vehicle control mice (*P* < 0.001, with nine mice per group, Fig. 2B and 2C). Importantly, the administration of Syn3 significantly mitigated RGC degeneration in DR mice (14.11 ± 1.54 vs. 20.22 ± 1.48 per view, *P* < 0.001, Fig. 2B and 2C). NeuN fluorescence staining of the retina section (Fig. 2D and 2E), confirmed the loss of NeuN-positive RGCs in GCL of DR mice (45.14% ± 9.17% of control mice, Fig. 2D and 2E). Remarkably, Syn3 administration significantly attenuated RGC degeneration in DR mice (Fig. 2D and 2E). The number of RGCs in the GCL (per view) was 9.78 ± 1.99 in DR mice, which increased to 14.11 ± 2.14 following Syn3 administration (random GCL views of nine mice per group, *P* < 0.001, Fig. 2D and 2E).

**Figure 2.**
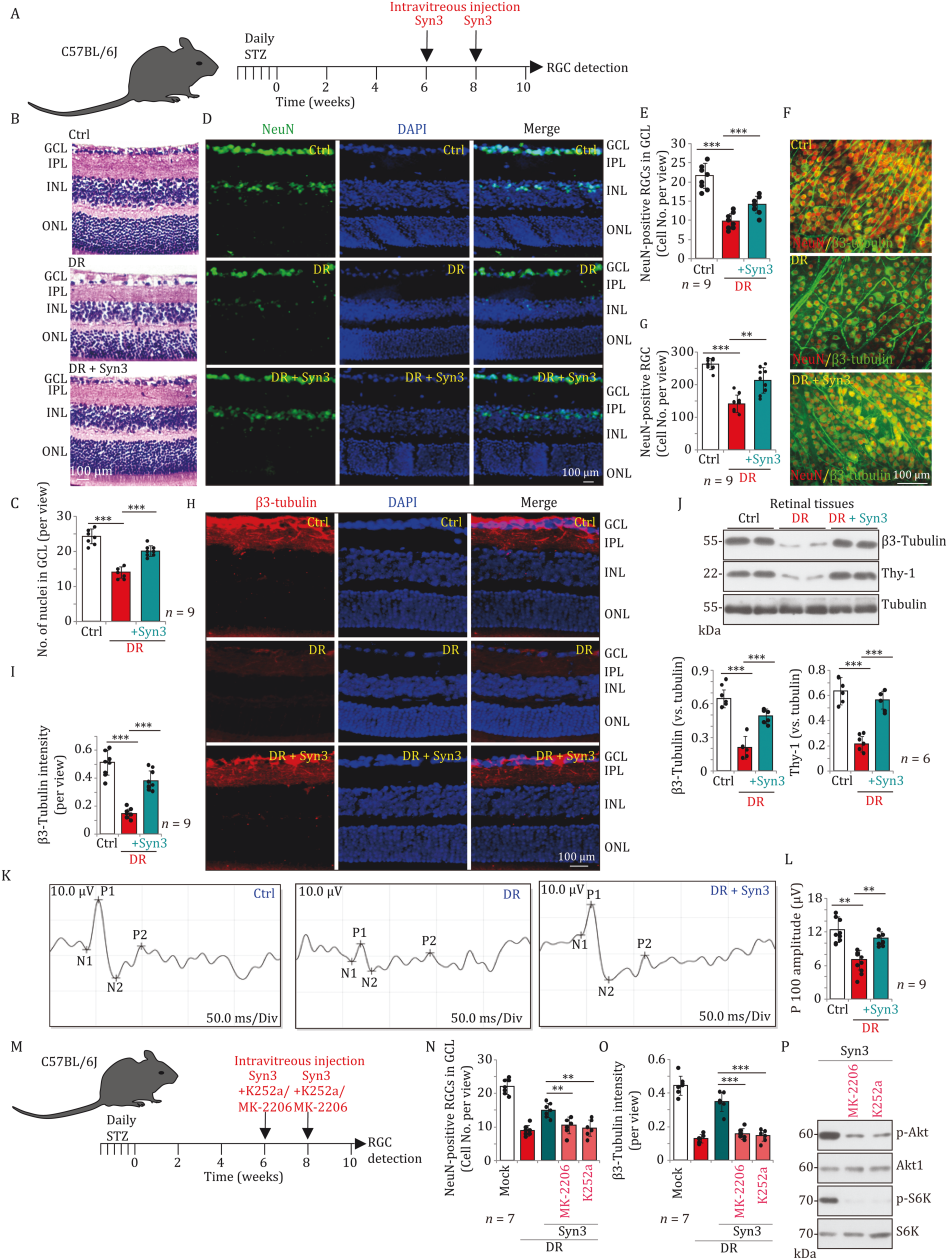
**Syn3 mitigates RGCs degeneration in DR mice.** DR mice were intravitreously injected with or without Syn3 (5 pmol in 0.5 μL saline) twice (A); 10 weeks after the last streptozotocin (STZ) administration, HE staining on paraffin-embedded retinal sections was performed and number of nuclei in GCL was quantified (B and C). NeuN/DAPI fluorescence staining in the retinal sections was shown and the number of NeuN-positive nuclei in GCL was quantified (D and E, scale bar = 100 μm). The fluorescence images of flat-mounted retinal GCL showing NeuN-/β3-tubulin-positive RGCs were presented and the number of RGCs was recorded (F and G). β3-Tubulin/DAPI fluorescence staining in the retinal slides was also shown and β3-tubulin fluorescence intensity was quantified (H and I). Expression of β3-tubulin and Thy-1 in retinal tissue lysates was tested by Western blot assays (J). The visual evoked potential (VEP) P100 amplitude was also recorded (F). DR mice were intravitreously injected with Syn3 (5 pmol), or plus MK-2205 (20 pmol)/K252a (2 pmol) twice (M); 10 weeks after the last streptozotocin (STZ) administration, NeuN/DAPI fluorescence staining in the retinal slides was carried out and the number of NeuN-positive nuclei in GCL was quantified (N). The retinal slides were also subjected to β3-tubulin/DAPI fluorescence staining, and β3-tubulin fluorescence intensity in GCL was quantified (O). Retinal tissues were collected and expression of listed proteins was shown (P). Data were presented as mean ± standard deviation (SD). *n* = 9 mice per group (B–I, K). *n* = 6 mice per group (J). *n* = 7 mice per group (N and O). **P < 0.01; ***P < 0.001. Scale bar = 100 μm. ONL, outer nuclear layer; INL, inner nuclear layer; IPL, inner plexiform layer.

Moreover, the flat-mounted GCL immunofluorescence assay demonstrated that the number of β3-tubulin-NeuN double-positive RGCs was significantly decreased in the retinas of DR mice (53.78% ± 10.22% of Ctrl mice, *P* < 0.001, Fig. 2F and 2G). Following Syn3 administration, the number of RGC staining for β3-tubulin and NeuN in DR mice was significantly increased (142.11 ± 27.00 vs. 214.22 ± 39.38 per view, random retinal views of nine mice per group, *P* < 0.001, Fig. 2F and 2G). Furthermore, the β3-tubulin fluorescence intensity was reduced in the GCL of DR mice (Fig. 2H and 2I), which was ameliorated by Syn3 administration (Fig. 2H and 2I). The expression of the RGC marker proteins, β3-tubulin and Thy-1, was decreased in retinal tissues from DR mice (Fig. 2J) and their expression was restored by Syn3 administration (Fig. 2J).

Using an electroretinogram (ERG) to measure the neuronal electrical activity of the retina in response to light, we found that the visual evoked potential (VEP) P100 amplitude in DR mice was reduced to 53.05% ± 15.94 % from that of control mice (*P* < 0.001, nine mice per group, Fig. 2K and 2L). Syn3 administration restored VEP (6.45 ± 1.94 μV vs. 10.64 ± 1.05 μV, *P* < 0.001, *n* = 9 mice per group, Fig. 2K and 2L), demonstrating that Syn3 maintained retinal function in DR mice. Importantly, the TrkB inhibitor K252a or the Akt inhibitor MK-2206 reversed Syn3-induced RGC protection in DR mice, showing that TrkB-Akt activation is required for the action of Syn3 (Fig. 2M). Syn3-induced inhibition of NeuN-positive RGC loss (Fig. 2N) and reduction in β3-tubulin fluorescence intensity (Fig. 2O) was reversed by co-administration of K252a or MK-2206. Western blot of retinal tissues confirmed that K252a and MK-2206 inhibited Akt and S6K phosphorylation in Syn3-treated mice (Fig. 2P).

Next, we examined whether the neuronal knockdown of Gαi1 and Gαi3 would lead to RGC degeneration. AAV9 viruses expressing Gαi1 shRNA and Gαi3 shRNA were intravitreously injected into C57B/6J mice, generating Gαi1 and Gαi3 neuronal double knockdown (“Gαi1/3-nDKD”) mice after 5 weeks (Fig. S5A). Control mice were intravitreously injected with AAV9-hSyn-scramble control shRNA (“shC”) virus. To confirm the specificity of the construct, an AAV9-hSyn-EGFP (enhanced green fluorescence protein) was intravitreously injected into C57B/6J mice and EGFP-positive staining was exclusively detected in GCL after 5 weeks (Fig. S5B).

In the retinal tissues of Gαi1/3-nDKD mice, *Gαi1* and *Gαi3* mRNA and protein levels were substantially decreased (*P* < 0.001 vs. shC mice, *n* = 5, Fig. S5C and S5D). *Gαi2* mRNA and protein expression in retinal tissues was not significantly changed. Gαi1/3-nDKD largely inhibited Akt and S6K phosphorylation in the retinal tissues (*P* < 0.001 vs. shC mice, *n* = 5, Fig. S5D). TrkB protein expression, and its phosphorylation and PSD95 protein expression were unchanged after Gαi1/3-nDKD (Fig. S5D). In Gαi1/3-nDKD mice, Syn3 administration (Fig. S5A) failed to alter *Gαi1*/*2*/*3* mRNA and protein expression (Fig. S5C and S5D), or affect TrkB-Akt-S6K phosphorylation in retinal tissues (Fig. S5D). These *in vivo* results further support the requirement of Gαi1/3 in Syn3-induced signaling.

Importantly, Gαi1/3-nDKD resulted in RGC degeneration. The number of NeuN-positive RGCs in the GCL of Gαi1/3-nDKD mice was 52.06% ± 8.92% of that in shC mice (quantifying random retinal views of nine mice per group, *P* < 0.001, Fig. S5E). Syn3 administration failed to attenuate RGCs degeneration in Gαi1/3-nDKD mice (Fig. S5E). The number of RGCs in GCL (per view) was 11.22 ± 1.92 in Gαi1/3-nDKD mice and was 10.78 ± 2.39 with Syn3 administration (quantifying random GCL views of nine mice per group, *P *> 0.05, Fig. S5E). Moreover, expression of the RGC marker proteins, Thy-1 and β3-tubulin, was decreased in retinal tissues of Gαi1/3-nDKD mice (quantifying retinal tissues from five mice per group, *P *< 0.001 vs. shC mice, Fig. S5F), which was again not prevented by Syn3 administration (*P *> 0.05, Fig. S5F). The quantified retinal flat mount RGC number results validated a decrease in TuJ1-positive RGCs in Gαi1/3-nDKD mice (quantifying random views of nine mice per group, *P* < 0.001, Fig. S5G). The administration of Syn3 failed to alleviate RGC degeneration in Gαi1/3-nDKD mice (with cell counts of 125.67 ± 13.37 vs. 127.00 ± 11.75, quantifying random views of nine mice per group, *P* > 0.05, Fig. S5G). Utilizing electroretinography (ERG) to assess the neuronal responses of the retina to light stimuli, the VEP P100 amplitude in Gαi1/3-nDKD mice decreased to 60.19% ± 9.37% compared to control mice (*P* < 0.001, with nine mice per group, Fig. S5H), and Syn3 administration did not significantly restore VEP amplitude (7.10 ± 1.11 μV vs. 6.89 ± 1.78 μV, *P* > 0.05, with nine mice per group, Fig. S5H).

The overexpression of Gαi1 and Gαi3 protein expression was found to augment the effects of Syn3 on BDNF signaling (Fig. S4). To examine if overexpression of Gαi1 and Gαi3 promotes the survival of RGCs *in vivo*, Gαi1 cDNA and Gαi3 cDNA sequences were individually inserted into the GV680 vector, and packed to generate AAV: AAV9-hSyn-Gαi1-OE and AAV9-hSyn-Gαi3-OE. Both viruses were intravitreously injected into adult mice (4-week old) and specifically increased Gαi1 and Gαi3 expression in RGCs (“Gαi1/3-nDOE”) after 5 weeks (Fig. S6A). As compared to vector (“Vec”) control mice, *Gαi1* and *Gαi3* mRNA (Fig. S6B) and protein (Fig. S6C) expression was significantly increased in the retinal tissues of Gαi1/3-nDOE mice, whereas *Gαi2* mRNA (Fig. S6B) and protein (Fig. S6C) expression was unchanged. Significantly, Gαi1/3-nDOE potently increased Akt and S6K phosphorylation in retinal tissue (Fig. S6C).

Exploring whether neuronal overexpression of Gαi1 and Gαi3 could prevent RGC degeneration in DR mice, 5 weeks after the last STZ administration, AAV9-hSyn-Gαi1-OE and AAV9-hSyn-Gαi3-OE were intravitreously injected into the mice, establishing Gαi1/3-nDOE after another 5 weeks (Fig. S6D). In STZ-administrated vector (“Vec”) mice, β3-tubulin and Thy-1 protein expression in retinal tissues was significantly decreased (Fig. S6E) but was mitigated in Gαi1/3-nDOE mice (Fig. S6E). These results demonstrate that neuronal overexpression of Gαi1 and Gαi3 can prevent RGC degeneration in DR mice, mimicking the actions of Syn3.

In the present study we demonstrate the neuroprotective efficacy of CN2097 and Syn3 in the retina through their ability to enhance TrkB signaling. In mouse and human primary RGCs, CN2097 significantly amplified BDNF-induced signaling, and in the STZ-induced DR mice, CN2097 mitigated RGC degeneration. In mouse and human primary RGCs, Syn3, which has a 5-fold higher affinity for the PSD95 PDZ3 domain, significantly increased BDNF-induced downstream signaling at one-tenth of the effective concentration of CN2097. Although CN2097 binds strongly to the PDZ3 domain of PSD95, it can also bind the PDZ1 domain, which may explain why it is less effective in stimulating BDNF signaling. Pretreatment of primary RGCs with Syn3 at 0.2 μmol/L, enhanced the BDNF-induced neuroprotection against OGD/R. Syn3 significantly increased RGC counts and inhibited apoptosis. These results were confirmed *in vivo* in the DR mouse model. Intravitreous injection of Syn3, at one-tenth, the concentration of CN2097, dramatically attenuated RGCs degeneration in DR mice and maintained retinal function.

We have established Gαi1/3 as pivotal signaling mediators in the activation of the Akt-mTOR signaling pathway by BDNF-TrkB. This study reveals that Gαi1/3 plays a crucial role as signaling mediators of the Syn3-enhancement of BDNF signaling. Syn3 treatment facilitated BDNF-induced formation of the TrkB-PSD95-Gαi1/3 complex, a pivotal step for initiating downstream Akt-mTOR signaling activation. Silencing of PSD95 inhibited the Syn3 facilitation of Akt-mTOR signaling following BDNF stimulation, underscoring the dependence of Syn3-enhanced BDNF signaling on PSD95. Similarly, Gαi1/3 gene silencing or DN mutations substantially inhibited downstream Akt-mTOR activation and suppressed the neuroprotective efficacy of Syn3. Conversely, overexpression of Gαi1/3 enhanced the activation of the downstream signaling pathways induced by Syn3 + BDNF. *In vivo*, neuronal silencing of Gαi1/3 significantly inhibited Akt-mTOR activation and reduced the number of RGCs. Notably, treatment with Syn3 did not increase Akt-mTOR activation or the number of RGCs within Gαi1/3-nDKD mice. In the DR model mice, neuronal overexpression of Gαi1/3 in RGCs enhanced Akt-mTOR activation and mitigated damage to RGCs. These results confirm the essential role of Gαi1/3 in Syn3-facilitated Akt-mTOR activation and associated neuroprotective effects.

This study shows the potential benefits of BDNF-enhancing compounds in mitigating diabetic retinal neurodegeneration. Our most promising compound, Syn3, exhibits high affinity and specificity for binding to the PDZ3 domain of PSD95. PDZ3 binding enhances the formation a TrkB-PSD95-Gαi1/3 complex in RGCs to enhance BDNF downstream signaling, thereby promoting survival. Our findings demonstrate that the development of drugs targeting the TrkB-PSD95-Gαi1/3 pathway offers a promising therapeutic approach for various conditions involving retinal neurodegeneration.

## Supplementary information

The online version contains supplementary material available at https://doi.org/10.1093/procel/pwae028.

pwae028_suppl_Supplementary_Materials

## Footnotes

This work was generously supported by the National Natural Science Foundation of China (81922025, 82371473, 82171080, 81970823, 82171461, 81771457, 82271107) and by Jiangsu Province Social Development Project (BE2023702).

This work was funded by the Priority Academic Program Development of Jiangsu Higher Education Institutions.

All authors conceived, designed, and supervised the study. All authors collected samples, performed experiments and analyzed the data, and were involved in drafting the article and revising it critically for important intellectual content. The authors declare on conflict of interest.

This study was approved by the Ethics Committee of Soochow University. All institutional and national guidelines for the care were carefully followed. All the authors agreed to participate in this paper and publish this manuscript.

All data generated or analyzed during this study are included in this published article (and its Supplementary information file).
