# Supplementary material for: Syn3, a newly developed cyclic peptide and BDNF signaling enhancer, ameliorates retinal ganglion cell degeneration in diabetic retinopathy
Source: Protein Cell. 2024 May 14;15(11):858–65. doi: 10.1093/procel/pwae028 (PMC11528515; doi:10.1093/procel/pwae028)
Supplement: pwae028_suppl_Supplementary_Materials [file pwae028_suppl_supplementary_materials.pdf]

Supplementary information

Syn3, a newly developed cyclic peptide and BDNF signaling enhancer, ameliorates retinal ganglion cell degeneration in diabetic retinopathy

This file includes:

Materials and methods

Figures S1-S6

Materials and methods

Chemical, reagents, RGCs and OGD/R procedure. CN2097 and Syn3 were from Dr. Marshall at Brown University (Naik et al., 2024). mRNA primers for Gαi1/2/3 were described early (Marshall et al., 2018; Shan et al., 2023; Xu et al., 2023). The viral constructs were sourced from Genechem (Shanghai, China), while all antibodies utilized in the study were acquired from Cell Signaling Technology (Danvers, MA). Comprehensive protocols for culturing primary murine RGCs and primary human RGCs have been documented in our prior studies (Tang et al., 2018; Chen et al., 2020). RGCs were cultured in Neurobasal plus medium (Thermo Fisher Scientific, Shanghai, China) supplemented with B27 supplement (Thermo Fisher Scientific) and antibiotics. The procedures of OGD/R on RGCs were reported in our previous studies (Chen et al., 2023; Liu et al., 2023). BDNF, insulin and PDGF-BB were provided by Sigma. Ethical approval for all research protocols involving human samples was granted by the Ethics Committee of Soochow University, ensuring adherence to the ethical principles outlined in the Helsinki Declaration.

Animals. All animal procedures were approved by the Ethics Committee and Institutional Animal Care and Use Committees (IACUC) of Soochow University (Suzhou, China). C57BL/6J mice, 4-5 week old, half male and half female, were purchased from Shanghai SLAC Laboratory Animal Co. Ltd (Shanghai, China). All mice received free access to food and water during the experiments in a specific-pathogen free (SPF) environment at constant temperature (20 ± 2°C) and constant humidity (55 ± 5%), maintained on a 12-h light/dark cycle.

Gene and protein detection. The comprehensive methodologies for co-immunoprecipitation (Co-IP), Western blotting, quantitative real-time PCR (qRT-PCR), and subsequent data analysis were extensively outlined in our earlier studies (Cao et al., 2009; Cao et al., 2013).

shRNA or gene overexpression *in vitro*. Lentiviral GV369 constructs harboring PSD95 shRNA (shPSD95-s1/shPSD95-s2 targeting non-overlapping sequences) or TrkB were obtained from Genechem (Shanghai, China). These constructs were individually transduced into HEK-293 cells using Lipofectamine 3000 to generate lentivirus. The resultant virus, with an MOI (multiplicity of infection) of 12, was introduced to cultured RGCs at DIV7. After six days, the

expression of PSD95/TrkB in RGCs at DIV13 was assessed through Western blot analysis. To silence Gαi1 and Gαi3, the Gαi1 shRNA-expressing lentivirus and the Gαi3 shRNA-expressing lentivirus, reported in our previous studies (Marshall et al., 2018; Shan et al., 2023; Xu et al., 2023), were co-added to primary RGCs for six days. The detailed protocols of overexpressing WT- or DN-Gαi1/3 were also reported in our previous studies (Marshall et al., 2018; Shan et al., 2023; Xu et al., 2023).

DR mouse model. As reported (Yao et al., 2022), C57B/6J mice, half male half female, 4-5 week old, underwent overnight fasting with ad libitum access to water. The following day, mice received intraperitoneal injections of 60 mg/kg freshly prepared streptozotocin (STZ), dissolved in cold 100 mM pH 4.5 citrate buffer (Sigma-Aldrich), for five consecutive days. One week after the final STZ injection, blood glucose levels were assessed using tail vein blood samples. Mice exhibiting fasting blood glucose levels exceeding 300 mg/dL (16.6 mmol/L) were identified as diabetic and included in subsequent investigations. Age-matched mice were administered with an equivalent volume of citrate buffer.

Intravitreal injection. The mice were anesthetized as described (Yao et al., 2022). Tropicamide eye drops were utilized to induce pupil dilation, while oxybuprocaine hydrochloride eye drops were utilized for ocular surface anesthesia. A 33G Hamilton needle was carefully inserted into the vitreous cavity, positioned 1 mm posterior to the corneoscleral limbus. Subsequently, 0.25/0.5 μL of solution (CN2097, Syn3, inhibitors or virus) was slowly injected into the vitreous cavity. Following completion of the injection, the needle remained in place within the vitreous cavity for an additional minute before being swiftly withdrawn. Methylcellulose eye drops were applied to maintain ocular moisture during the surgical procedure, while ofloxacin eye drops were administered to the operated eye post-surgery to prevent infection. Under anesthesia, all mice were euthanized via cervical dislocation, following which retinas were carefully excised for further analysis.

Neuronal knockdown of Gαi1 and Gαi3 *in vivo*. The GV680 shRNA plasmids designed to target Gαi1 (*GNAI1*) or Gαi3 (*GNAI3*) under the hSyn promoter of adeno-associated virus 9 (AAV9) (as reported early (Chen et al., 2023)) were provided by Genechem (Shanghai, China). These constructs were separately transfected into HEK-293 cells to produce AAV, which were subsequently intravitreally administered to the mice following established protocols. Control

mice received injection of AAV9-hSyn scramble control shRNA (“shC”).

Gαi1/3-overexpressing AAV. The Gαi1 cDNA (NM_010305.1) sequence or the Gαi3 cDNA (NM_010306.3) sequence was inserted into *Eco*RI and *BamHI* site of the hSyn promoter-MIR155(MCS)-SV40 PolyA construct (GV680 without EGFP, as reported early (Chen et al., 2023). The construct along with the AAV9 packaging system (Genechem) were co-transfected to HEK-293 cells, generating AAV9-hSyn-Gαi1-OE and AAV9-hSyn-Gαi3-OE. The titers of AAV9 were approximately to 1.0×10^{12} TU/mL, and were subsequently intravitreally administered to the mice following established protocols..

Hematoxylin and eosin (HE) staining. The retinal tissues were immersed in 4% paraformaldehyde (PFA) for a duration of 12h, followed by embedding in paraffin and subsequent sectioning. Sections oriented parallel to the eye axis were obtained. Hematoxylin and eosin (HE) staining was then conducted. The number of nuclei within GCL was observed and captured under a light microscope.

Retinal frozen section fluorescence staining. As described (Yao et al., 2022; Shan et al., 2023; Xu et al., 2023), The excised retinal tissue was fixed using a 4% paraformaldehyde (PFA) solution overnight, followed by dehydration in 30% sucrose for another day. The retinal tissues were then embedded in OCT embedding Matrix (Fisher Scientific) and rapidly frozen. Subsequently, 10 μm sections were sliced at -25 ℃. These sections were incubated in a 5% bovine serum albumin (BSA) solution (Sigma) in PBS containing 0.5% Triton X-100 (PBST) at 37 ℃ for 1h. Next, sections were treated with primary antibodies anti-NeuN (1:1000; Abcam) or anti-β3 Tubulin (1:1000; Abcam) overnight at 4 ℃. The frozen slides were then rinsed with PBST the following day and incubated with secondary antibodies (1:500; Abcam) for 6h at room temperature in the dark. Finally, the sections were incubated with DAPI for 10 min, washed, and captured under a confocal laser microscope.

Retinal flat mount fluorescence staining. Retinal flat mounts underwent fixation with 4% PFA for 12 min at room temperature. Subsequently, they were immersed in a 5% BSA solution in PBST for 40 min at 37 ℃. Following this, sections were exposed to primary antibodies, including anti-NeuN, anti-β3 tubulin and anti-Tuj1 (1:1000; Abcam), and left to incubate overnight at 4 °C. On the subsequent day, the flat mounts were rinsed with PBST and then

treated with the secondary antibody (1:500; Abcam) for 3h at room temperature in darkness. Finally, the flat mounts were examined and photographed under a confocal microscope.

ERG. As described (Yan et al., 2014), the mice underwent overnight dark adaptation followed by full dilation of their pupils using a 1% tropicamide solution (Alcon, Fort Worth, TX). ERG responses were recorded from both eyes using platinum wire corneal electrodes, a forehead reference electrode, and a tail-mounted ground electrode. ERG waveforms were captured with a bandwidth spanning 0.3 to 500 Hz and sampled at 2 kHz through a digital acquisition system. The amplitudes of a-, b- waves were recorded.

Other cellular methods, including nuclear TUNEL staining, medium LDH releasing assay, CCK-8 cell viability, cellular fluorescence staining were described in detail in our previous studies (Chen et al., 2023; Liu et al., 2023).

Statistical analysis. Data were all with normal distribution and were presented as mean ± standard deviation (SD). The two-tailed unpaired t test was utilized to compare statistical difference between two specific groups. One-way analysis of variance (ANOVA) plus Tukey's post hoc test were utilized for multiple groups. P values < 0.05 was statistically significant.

Availability of data and material. All data generated or analyzed during this study are included in this published article [and its supplementary information files].

References

Cao, C., Huang, X., Han, Y., Wan, Y., Birnbaumer, L., Feng, G.S., Marshall, J., Jiang, M., and Chu, W.M. (2009). Galpha(i1) and Galpha(i3) are required for epidermal growth factor-mediated activation of the Akt-mTORC1 pathway. Sci Signal 2, ra17.

Cao, C., Rioult-Pedotti, M.S., Migani, P., Yu, C.J., Tiwari, R., Parang, K., Spaller, M.R., Goebel, D.J., and Marshall, J. (2013). Impairment of TrkB-PSD-95 signaling in Angelman syndrome. PLoS Biol 11, e1001478.

Chen, N., Li, Y., Huang, N., Yao, J., Luo, W.F., and Jiang, Q. (2020). The Nrf2 activator MIND4-17 protects retinal ganglion cells from high glucose-induced oxidative injury. J Cell Physiol 235, 7204-7213.

Chen, Z.G., Shi, X., Zhang, X.X., Yang, F.F., Li, K.R., Fang, Q., Cao, C., Chen, X.H., and Peng, Y. (2023). Neuron-secreted NLGN3 ameliorates ischemic brain injury via activating Galphai1/3-Akt signaling. Cell Death Dis 14, 700.

Liu, T.T., Shi, X., Hu, H.W., Chen, J.P., Jiang, Q., Zhen, Y.F., Cao, C., Liu, X.W., and Liu, J.G. (2023). Endothelial cell-derived RSPO3 activates Galphai1/3-Erk signaling and protects neurons from ischemia/reperfusion injury. Cell Death Dis 14, 654.

Marshall, J., Zhou, X.Z., Chen, G., Yang, S.Q., Li, Y., Wang, Y., Zhang, Z.Q., Jiang, Q., Birnbaumer, L., and Cao, C. (2018). Antidepression action of BDNF requires and is mimicked by Galphai1/3 expression in the hippocampus. Proc Natl Acad Sci U S A 115, E3549-E3558.

Naik, M.T., Naik, N., Hu, T., Wang, S.H., and Marshall, J. (2024). Structure-based design of peptidomimetic inhibitors of PSD-95 with improved affinity for the PDZ3 domain. FEBS Lett 598, 233-241.

Shan, H.J., Jiang, K., Zhao, M.Z., Deng, W.J., Cao, W.H., Li, J.J., Li, K.R., She, C., Luo, W.F., Yao, J., *et al.* (2023). SCF/c-Kit-activated signaling and angiogenesis require Galphai1 and Galphai3. Int J Biol Sci 19, 1910-1924.

Tang, C.Z., Li, K.R., Yu, Q., Jiang, Q., Yao, J., and Cao, C. (2018). Activation of Nrf2 by Ginsenoside Rh3 protects retinal pigment epithelium cells and retinal ganglion cells from UV. Free Radic Biol Med 117, 238-246.

Xu, G., Qi, L.N., Zhang, M.Q., Li, X.Y., Chai, J.L., Zhang, Z.Q., Chen, X., Wang, Q., Li, K.R., and Cao, C. (2023). Galphai1/3 mediation of Akt-mTOR activation is important for RSPO3-induced angiogenesis. Protein Cell 14, 217-222.

Yan, B., Tao, Z.F., Li, X.M., Zhang, H., Yao, J., and Jiang, Q. (2014). Aberrant expression of long noncoding RNAs in early diabetic retinopathy. Invest Ophthalmol Vis Sci 55, 941-951.

Yao, J., Wu, X.Y., Yu, Q., Yang, S.F., Yuan, J., Zhang, Z.Q., Xue, J.S., Jiang, Q., Chen, M.B., Xue, G.H., *et al.* (2022). The requirement of phosphoenolpyruvate carboxykinase 1 for angiogenesis in vitro and in vivo. Sci Adv 8, eabn6928.

Figure S1.

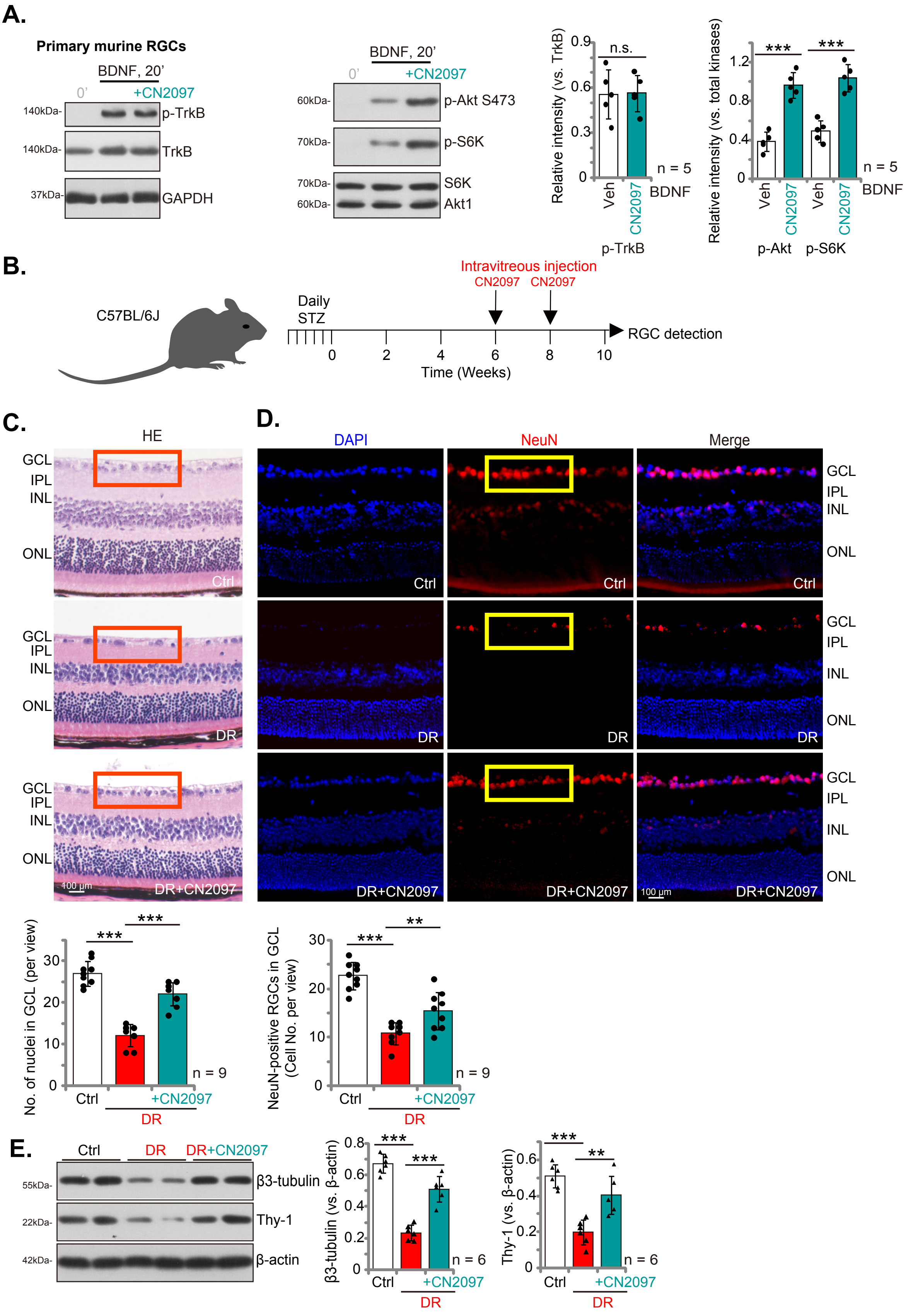

A.
Primary murine RGCs
BDNF, 20'
0'
+CN2097
140kDa-
p-TrkB
140kDa-
TrkB
37kDa-
GAPDH
BDNF, 20'
0'
+CN2097
60kDa-
p-Akt S473
70kDa-
p-S6K
70kDa-
S6K
60kDa-
Akt1
Relative intensity (vs. TrkB)
n.s.
n = 5
Veh
CN2097
BDNF
p-TrkB
Relative intensity (vs. total kinases)
***
***
n = 5
Veh
CN2097
Veh
CN2097
BDNF
p-Akt
p-S6K
B.
C57BL/6J
Daily
STZ
Intravitreous injection
CN2097
CN2097
RGC detection
Time (Weeks)
C.
HE
GCL
IPL
INL
ONL
Ctrl
DR
DR+CN2097
400 μm
D.
DAPI
NeuN
Merge
Ctrl
DR
DR+CN2097
100 μm
No. of nuclei in GCL (per view)
***
***
n = 9
Ctrl
+CN2097
DR
NeuN-positive RGCs in GCL
(Cell No. per view)
***
**
n = 9
E.
Ctrl
DR
DR+CN2097
55kDa-
β3-tubulin
22kDa-
Thy-1
42kDa-
β-actin
β3-tubulin (vs. β-actin)
***
***
n = 6
Thy-1 (vs. β-actin)
***
**
n = 6

Figure S2.

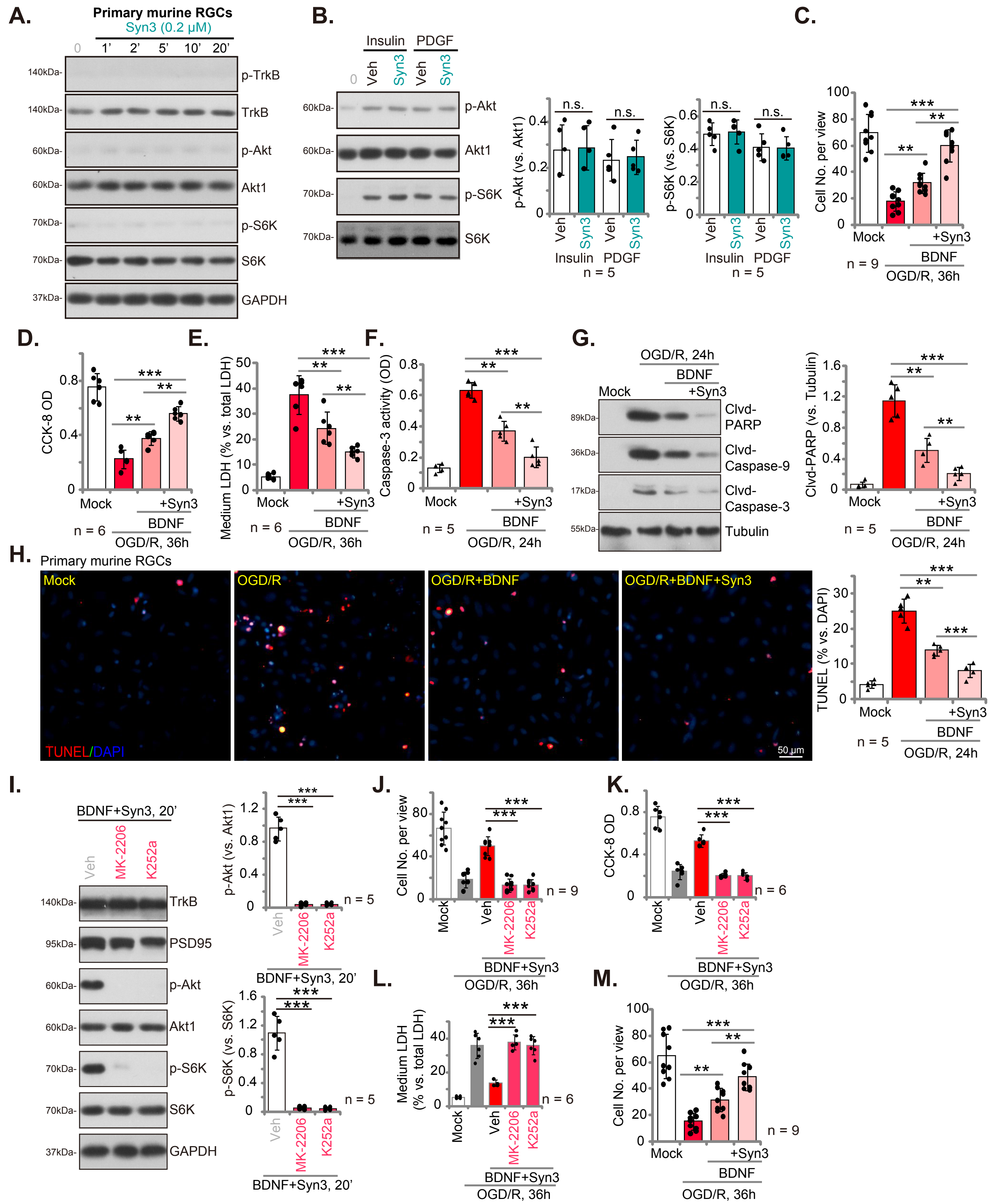
A.
Primary murine RGCs
Syn3 (0.2 μM)
0 1' 2' 5' 10' 20'
p-TrkB
TrkB
p-Akt
Akt1
p-S6K
S6K
GAPDH
B.
Insulin PDGF
0 Veh Syn3 Veh Syn3
p-Akt
Akt1
p-S6K
S6K
n.s.
p-Akt (vs. Akt1)
p-S6K (vs. S6K)
Insulin PDGF
n = 5
C.
Cell No. per view
Mock
+Syn3
BDNF
OGD/R, 36h
n = 9
D.
CCK-8 OD
n = 6
E.
Medium LDH (% vs. total LDH)
F.
Caspase-3 activity (OD)
n = 5
OGD/R, 24h
G.
OGD/R, 24h
BDNF
Mock
+Syn3
Clvd-PARP
Clvd-Caspase-9
Clvd-Caspase-3
Tubulin
Clvd-PARP (vs. Tubulin)
H.
Primary murine RGCs
Mock
OGD/R
OGD/R+BDNF
OGD/R+BDNF+Syn3
TUNEL/DAPI
50 μm
TUNEL (% vs. DAPI)
I.
BDNF+Syn3, 20'
Veh MK-2206 K252a
TrkB
PSD95
p-Akt
Akt1
p-S6K
S6K
GAPDH
p-Akt (vs. Akt1)
p-S6K (vs. S6K)
J.
K.
L.
M.
BDNF+Syn3
OGD/R, 36h
Medium LDH (% vs. total LDH)

Figure S3

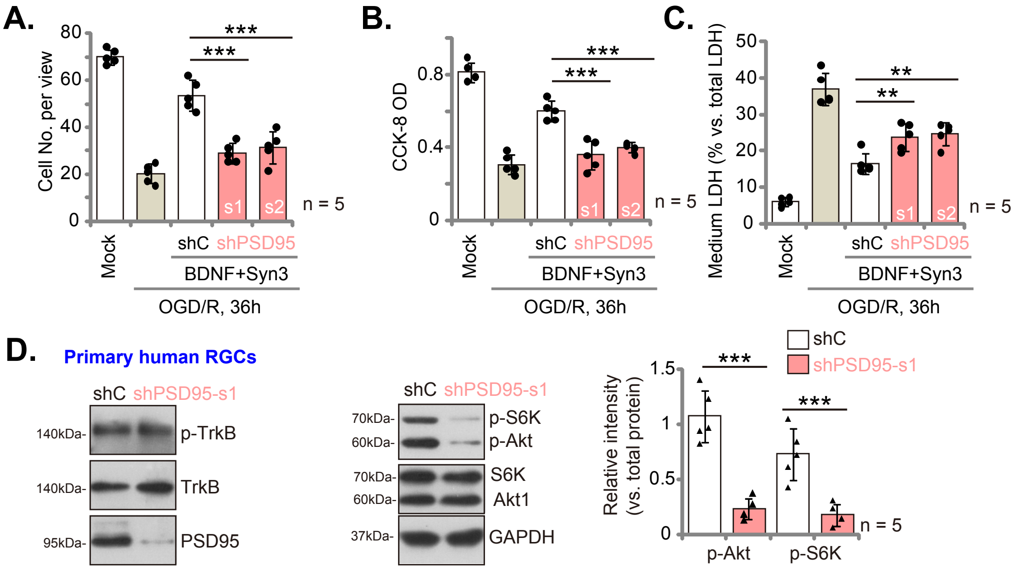

A.
Cell No. per view
80
60
40
20
0
***
***
s1
s2
n = 5
Mock
shC shPSD95
BDNF+Syn3
OGD/R, 36h
B.
CCK-8 OD
0.8
0.4
0
***
***
s1
s2
n = 5
Mock
shC shPSD95
BDNF+Syn3
OGD/R, 36h
C.
Medium LDH (% vs. total LDH)
50
40
30
20
10
0
**
**
s1
s2
n = 5
Mock
shC shPSD95
BDNF+Syn3
OGD/R, 36h
D.
Primary human RGCs
shC shPSD95-s1
140kDa- p-TrkB
140kDa- TrkB
95kDa- PSD95
shC shPSD95-s1
70kDa- p-S6K
60kDa- p-Akt
70kDa- S6K
60kDa- Akt1
37kDa- GAPDH
shC
shPSD95-s1
Relative intensity (vs. total protein)
1.5
1
0.5
0
***
***
n = 5
p-Akt
p-S6K

Figure S4.

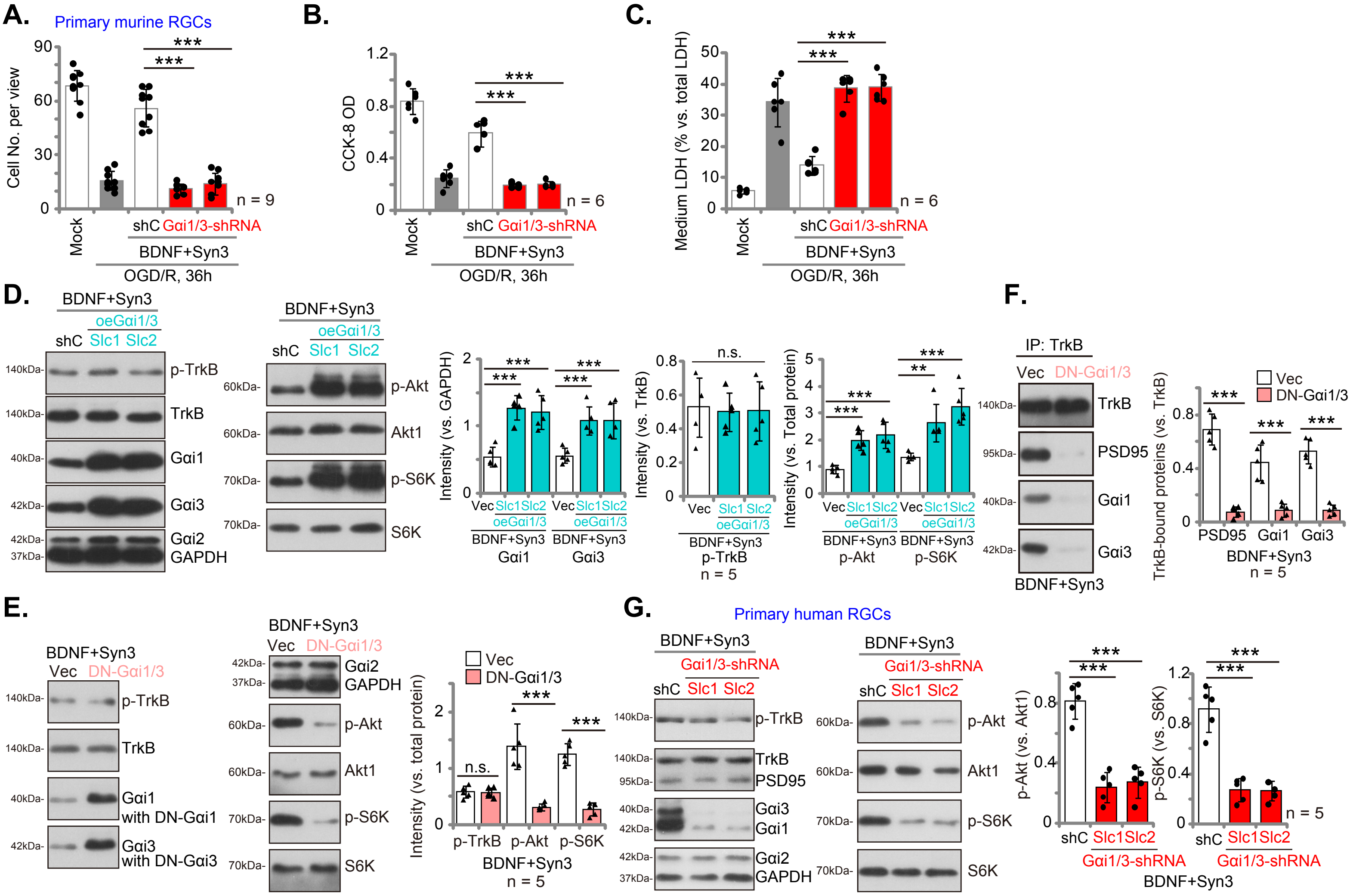

A.
Primary murine RGCs
Cell No. per view
Mock
shC
Gαi1/3-shRNA
BDNF+Syn3
OGD/R, 36h
n = 9
B.
CCK-8 OD
n = 6
C.
Medium LDH (% vs. total LDH)
n = 6
D.
BDNF+Syn3
oeGαi1/3
shC
Slc1
Slc2
p-TrkB
TrkB
Gαi1
Gαi3
Gαi2
GAPDH
p-Akt
Akt1
p-S6K
S6K
Intensity (vs. GAPDH)
Intensity (vs. TrkB)
Intensity (vs. Total protein)
Vec
n.s.
n = 5
E.
DN-Gαi1/3
Gαi1 with DN-Gαi1
Gαi3 with DN-Gαi3
Intensity (vs. total protein)
F.
IP: TrkB
PSD95
TrkB-bound proteins (vs. TrkB)
G.
Primary human RGCs
p-Akt (vs. Akt1)
p-S6K (vs. S6K)

Figure S5.

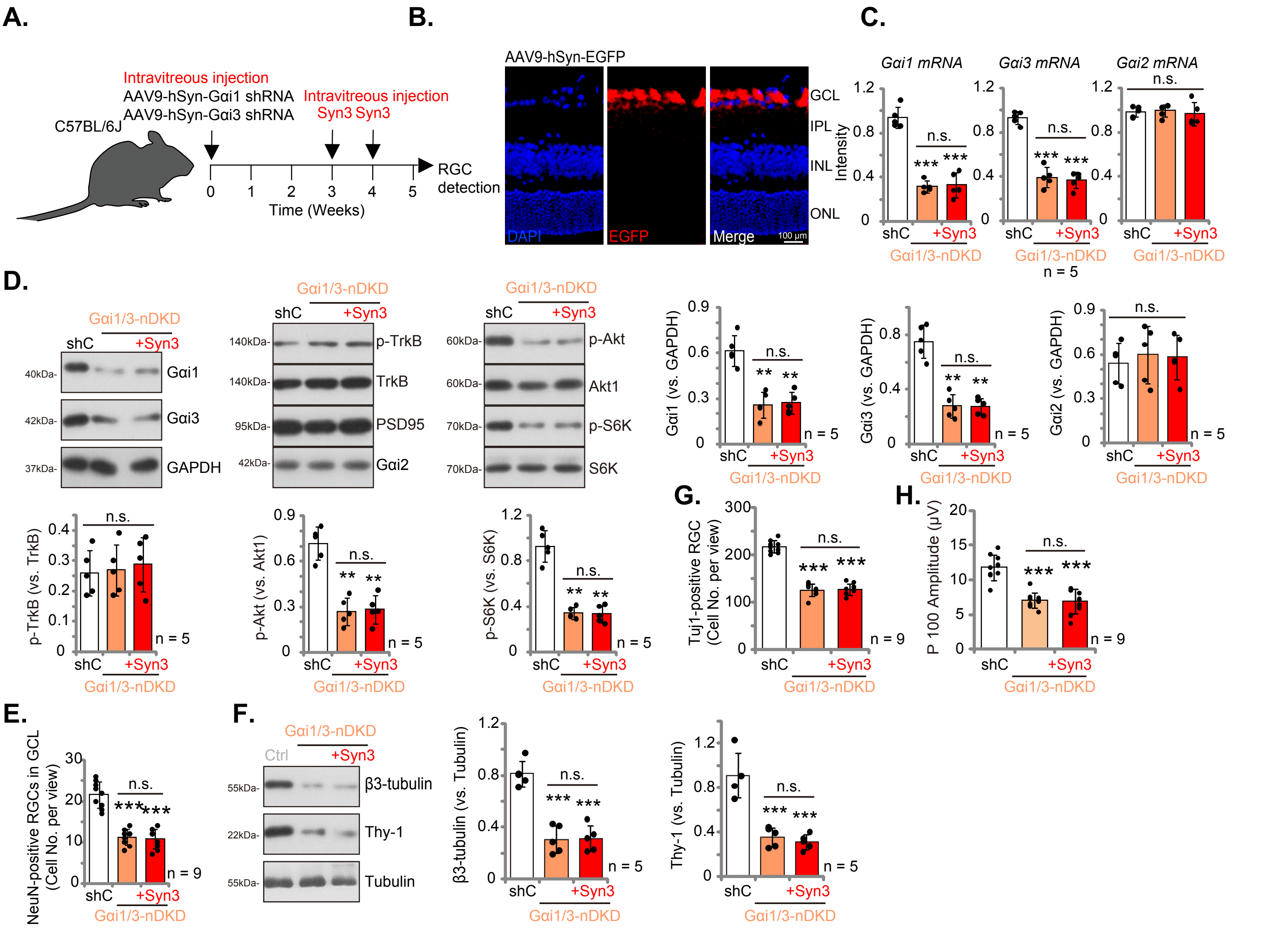
A.
Intravitreous injection
AAV9-hSyn-Gαi1 shRNA
AAV9-hSyn-Gαi3 shRNA
Intravitreous injection
Syn3 Syn3
C57BL/6J
RGC detection
Time (Weeks)
B.
AAV9-hSyn-EGFP
DAPI
EGFP
Merge
GCL
IPL
INL
ONL
100 μm
C.
Gαi1 mRNA
Gαi3 mRNA
Gαi2 mRNA
Intensity
shC
+Syn3
Gαi1/3-nDKD
n = 5
D.
Gαi1
Gαi3
GAPDH
p-TrkB
TrkB
PSD95
Gαi2
p-Akt
Akt1
p-S6K
S6K
Gαi1 (vs. GAPDH)
Gαi3 (vs. GAPDH)
Gαi2 (vs. GAPDH)
p-TrkB (vs. TrkB)
p-Akt (vs. Akt1)
p-S6K (vs. S6K)
G.
Tuj1-positive RGC (Cell No. per view)
n = 9
H.
P 100 Amplitude (μV)
E.
NeuN-positive RGCs in GCL (Cell No. per view)
F.
Ctrl
β3-tubulin
Thy-1
Tubulin
β3-tubulin (vs. Tubulin)
Thy-1 (vs. Tubulin)

Figure S6.

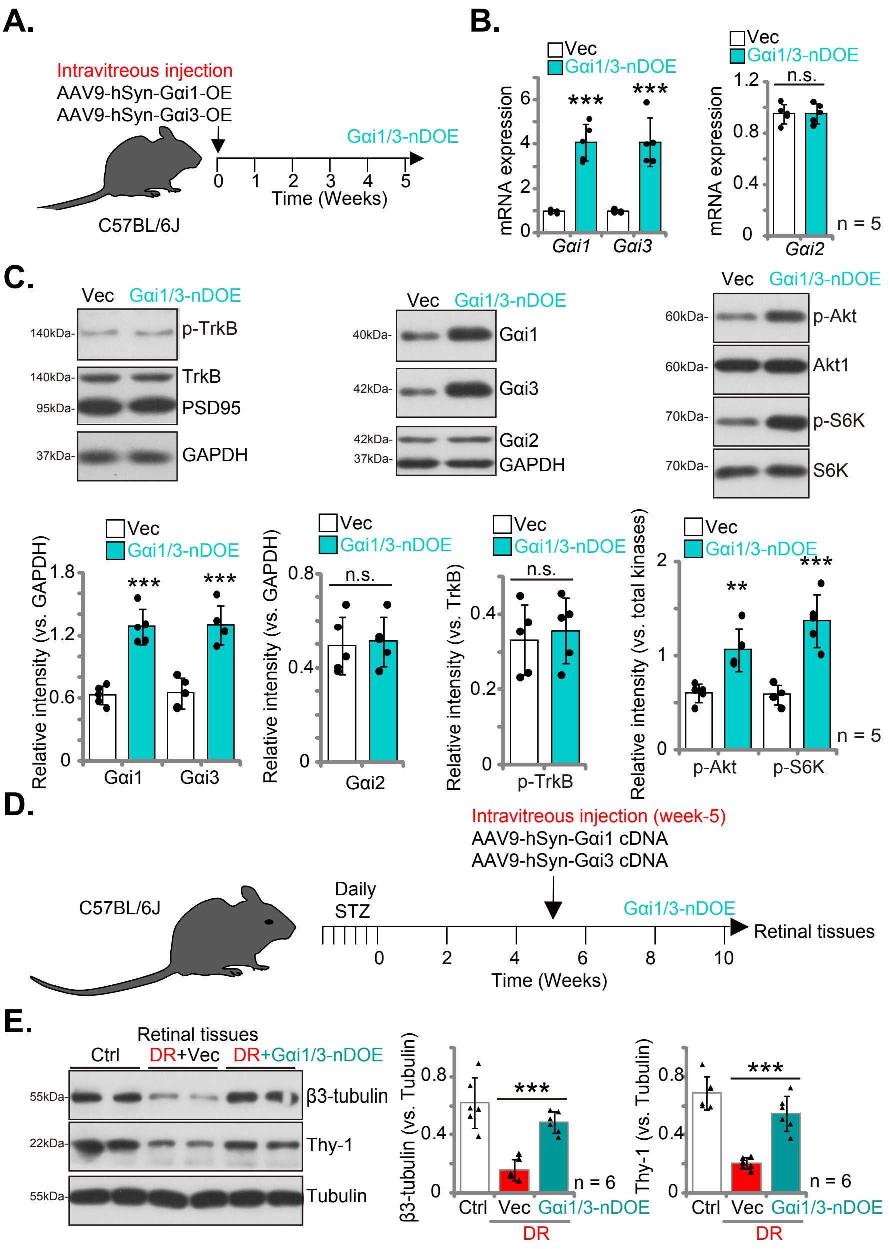
A.
Intravitreous injection
AAV9-hSyn-Gαi1-OE
AAV9-hSyn-Gαi3-OE
Gαi1/3-nDOE
Time (Weeks)
C57BL/6J
B.
Vec
Gαi1/3-nDOE
mRNA expression
Gαi1
Gαi3
Gαi2
n.s.
n = 5
C.
p-TrkB
TrkB
PSD95
GAPDH
Gαi1
Gαi3
Gαi2
p-Akt
Akt1
p-S6K
S6K
Relative intensity (vs. GAPDH)
Relative intensity (vs. TrkB)
Relative intensity (vs. total kinases)
p-Akt
p-S6K
D.
Intravitreous injection (week-5)
AAV9-hSyn-Gαi1 cDNA
AAV9-hSyn-Gαi3 cDNA
Daily
STZ
Retinal tissues
E.
Retinal tissues
Ctrl
DR+Vec
DR+Gαi1/3-nDOE
β3-tubulin
Thy-1
Tubulin
β3-tubulin (vs. Tubulin)
Thy-1 (vs. Tubulin)
DR
n = 6

Figure S1. TrkB-enhancer CN2097 mitigates RGC degeneration in DR mice. The primary murine RGCs were pretreated with CN2097 (2 μM) or vehicle control (saline, "Veh") for 20 min, followed by BDNF stimulation (25 ng/mL) for another 20 min, listed proteins in total cell lysates were measured (**A**). DR mice were intravitreously injected with or without CN2097 (50 pmol in 0.5μL saline) two times (**B**); Ten weeks after the last streptozotocin (STZ) administration, hematoxylin and eosin (HE) staining on paraffin-embedded retinal sections was performed and number of nuclei in GCL was quantified (**C**). NeuN/DAPI fluorescence staining in the retinal sections of the mice was also shown and number of NeuN-positive nuclei in retinal ganglion cell layer (GCL) was quantified (**D**). Expression of listed proteins in retinal lysates was also tested (**E**). Data were presented as mean ± standard deviation (SD). n = 5/6 stands for five/six biological repeats (**A** and **E**). n = 9 mice per group (**C**-**D**). *** $P < 0.001$. ** $P < 0.001$. "n.s." stands for non-statistical difference ($P > 0.05$). Scale bar = 100 μm. GCL: Ganglion Cell Layer, ONL: Outer Nuclear Layer, INL: Inner Nuclear Layer, IPL: Inner Plexiform Layer.

Figure S2. Syn3 potentiates BDNF-induced RGC protection against oxygen glucose deprivation/re-oxygenation. The primary murine RGCs were treated with Syn3 (0.2 μM) only for indicated time periods and listed protein were tested (**A**). The primary murine RGCs were pretreated with Syn3 (0.2 μM) or vehicle control (saline, "Veh") for 20 min, followed by insulin (1 μg/mL) or PDGF (25 ng/mL) treatment for another 20 min, expression of listed proteins in total cell lysates was shown (**B**). The primary murine RGCs were pre-treated with Syn3 (0.2 μM) or PBS vehicle control ("Veh") for 20 min, followed by BDNF (25 ng/mL) for another 20 min, cells were subjected to 4h of OGD plus 24/36h of re-oxygenation (OGD/R); Cell number (**C**), viability (**D**) and medium LDH release (**E**) were measured; Cell apoptosis was valued by the described methods (**F**-**H**). The primary murine RGCs were treated with the TrkB inhibitor K252a (200 nM) or the pan Akt inhibitor MK-2206 (20 μM) for 30', Syn3 (0.2 μM) was thereafter added for 20 min and BDNF (25 ng/mL) was added afterwards ("BDNF+Syn3"); Twenty minutes (20 min) after BDNF stimulation, expression of listed proteins in total cellular lysates was shown (**I**); Alternatively, murine RGCs were subjected to OGD/R stimulation for 36h, cell number (**J**), viability (**K**) and medium LDH release (**L**) were tested. The primary human RGCs were pre-treated with Syn3 (0.2 μM) or PBS vehicle control ("Veh") for 20 min, followed by BDNF (25 ng/mL) for another 20 min, cells were subjected to 4h of OGD plus 36h of re-oxygenation (OGD/R), cell number was recorded (**M**). Data were presented as mean ±

standard deviation (SD). n = 5/9 stands for five/nine biological repeats. "n.s." stands for non-statistical difference ($P > 0.05$). ** $P < 0.01$; *** $P < 0.001$. Scale bar = 50 μm.

Figure S3. Syn3 facilitates TrkB-PSD95-Gαi1/3 complex formation in RGCs. The primary murine RGCs with the listed lentiviral PSD95 shRNA ("shPSD95-s1" or "shPSD95-s2") or the lentiviral scramble control shRNA ("shC") were treated with BDNF (25 ng/mL) plus Syn3 (0.2 μM, 20 min pretreatment) ("BDNF+Syn3"), and were further subject to OGD/R stimulation and cultivated for another 36h, cell number (per view, **A**), viability (CCK-8 OD, **B**) and death (by measuring medium LDH release, **C**) were tested. The primary human RGCs with the lentiviral PSD95 shRNA ("shPSD95-s1") or "shC" were treated with BDNF+Syn3 for 20 min and were analyzed for the listed proteins in total cellular lysates (**D**); Data were presented as mean ± standard deviation (SD). ** $P < 0.01$, *** $P < 0.001$. n = 5 stands for five biological repeats.

Figure S4. Gαi1 and Gαi3 play pivotal roles in Syn3-induced enhancement of TrkB signaling. The primary murine RGCs with the lentiviral Gαi1 shRNA plus lentiviral Gαi3 shRNA (Gαi1/3-shRNA) or the lentiviral scramble control shRNA (shC) were treated with BDNF (25 ng/mL) plus Syn3 (0.2 μM, 20 min pretreatment) ("BDNF+Syn3"), and were subjected to ODG/R stimulation for another 36h, cell number (per view, **A**), viability (CCK-8 OD, **B**) and death (by measuring medium LDH release, **C**) were tested. The primary murine RGCs with the lentivirus-packed Gαi1-expressing construct and the lentivirus-packed Gαi3-expressing construct (oeGαi1/3, "Slc1/Slc2" stands for two cell selection) or the empty vector ("Vec") were treated with BDNF (25 ng/mL) plus Syn3 (0.2 μM, 20 min pretreatment) ("BDNF+Syn3") and were analyzed for the listed proteins in total cellular lysates (**D**); The primary murine RGCs with the lentivirus-packed dominant negative (DN)-Gαi1 construct and the lentivirus-packed DN-Gαi3 construct (DN-Gαi1/3) or the empty vector ("Vec") were treated with BDNF+Syn3 and were analyzed for the listed proteins in total cellular lysates (**E**). TrkB-associated PSD95, Gαi1 and Gαi3 were tested via co-immunoprecipitation assays (**F**). The primary human RGCs with Gαi1/3-shRNA or shC were treated with BDNF+Syn3 for 20', expression of listed proteins in total cell lysates was tested (**G**). Data were presented as mean ± standard deviation (SD). *** $P < 0.001$. "n.s." stands for non-statistical difference ($P > 0.05$). n = 5-9 stands for biological repeats.

Figure S5. Neuronal knockdown of Gαi1 and Gαi3 induces RGC degeneration in retinas, not rescued by Syn3. C57B/6J adult mice (4-5-week) were intravitreously injected with the AAV9-hSyn-Gαi1 shRNA plus the AAV9-hSyn-Gαi3 shRNA (0.25 μL each, Gαi1/3-nDKD) and were further intravitreously injected with or without Syn3 (5 pmol in 0.5 μL saline, twice); Control mice were intravitreously injected with AA9-hSyn3 scramble control shRNA ("shC") (**A**) or AA9-hSyn3-EGFP (GV680 construct) (**B**). Retinal tissues were obtained and expression of listed mRNAs (**C**) and proteins (**D** and **F**) were tested. NeuN/DAPI fluorescence staining in the retinal slides was shown and the number of NeuN-positive nuclei in GCL was quantified (**E**). The fluorescence images of flat-mounted retinal GCL showing NeuN-/β3-tubulin-positive RGCs were performed and the number of RGCs per view was recorded (**G**); The visual evoked potential (VEP) P100 amplitude was also recorded, with results quantified (**H**). Data were presented as mean ± standard deviation (SD). n = 5 stands for tissues of five mice per group (**C**, **D** and **F**). n = 9 mice per group (**E**-**H**). ** $P < 0.01$, *** $P < 0.001$ *vs.* shC group. "n.s." stands for non-statistical difference ($P > 0.05$). Scale bar = 100 μm. ONL: Outer Nuclear Layer, INL: Inner Nuclear Layer, IPL: Inner Plexiform Layer.

Figure S6. Neuronal overexpression of Gαi1 and Gαi3 inhibits RGCs degeneration in DR mice. The C57B/6J adult mice (4-week old) were intravitreously injected with AAV9-hSyn-Gαi1-expressing GV680 construct ("AAV9-hSyn-Gαi1-OE", 0.25 μL) plus AAV9-hSyn-Gαi3-expressing GV680 construct ("AAV9-hSyn-Gαi3-OE",0.25 μL) ("Gαi1/3-nDOE") (**A**). Control mice were administrated with the empty AA9-hSyn3-GV680 construct (0.5μL) ("Vec"). After five weeks, expression of listed mRNAs and proteins in retinal tissues was shown (**B** and **C**). STZ-administrated DR mice were also subjected to the same "Gαi1/3-nDOE" or "Vec" procedure (**D**), 10 weeks after the last STZ administration, β3-tubulin and Thy-1 protein expression in retinal tissues was examined and results were quantified (**E**). Data were presented as mean ± standard deviation (SD). n = 5 stands for five biological repeats (**B** and **C**). n = 6 mice per group (**E**). ** $P < 0.01$, *** $P < 0.001$ *vs.* "Ctrl" group (**B** and **C**). *** $P < 0.001$ (**E**). "n.s." stands for non-statistical difference ($P > 0.05$).
